# An analysis of *Sorghum halepense*’s behavior in presence of tropane alkaloids from *Datura stramonium* extracts

**DOI:** 10.1186/1752-153X-6-75

**Published:** 2012-07-28

**Authors:** Monica Butnariu

**Affiliations:** 1 Chemistry and Biochemistry Department, Banat's University of Agricultural Sciences and Veterinary Medicine from Timisoara, Calea Aradului, no. 119, Timisoara, 300645, Romania

## Abstract

**Background:**

This study aimed to quantify the allelopathic potential of *Datura stramonium* (Jimson weed). *Sorghum halepense* (Johnsongrass) tolerance was assessed by germinating, seed and growing seedlings, dosing of photo-synthesis pigments, followed by treatment with *D. stramonium* extract tropane alkaloids.

**Results:**

Preliminary chemical analysis of the extracts showed the presence of alkaloids.

The presence of alkaloids was confirmed through HPLC–UV system analysis. Various concentrations of analytic purity alkaloids had similar effects on germination and development of *S. halepense’*s root systems with those of extracts from of *D. stramonium.* Germination was not affected by any of the tested extracts, but growth was inhibited by the presence of tropane alkaloids. Extracts had effects at higher alkaloid concentrations. Seedlings of *S. halepense* developed toxicity symptoms in the presence of alkaloid extracts, but the occurrence of several chlorotic and necrotic areas was noticed in the flower extract biotest.

**Conclusions:**

Results show that the tested species is sensitive to alkaloids in their growth environment. This research justifies the fact that aqueous extracts from *D. stramonium* are adequate to the situations in which *S. halepense* becomes damaging.

## Background

The approach and solutions suggested in this study include a complex comparative analysis of the behaviour of *S. halepense* plants and a combination of some relatively traditional methods (germination and seedling growth) [1] and chemical methods. A research focused on a set of results pointing to useful information aiming to controlling the pest species [2] requires a complex, multidisciplinary approach [3] in line with the latest achievements [4]. The nature of genetic resources shows that there is a high genetic variability of selections and ecological solutions, which have the potential to reach performances in the ecological agriculture system.[5-7].

Interactions between species are part of the characteristic biocoenosis structure [8,9].

Some plants release toxic substances that inhibit the growth of other plants (allelopathy), a feature that represents a competitive advantage [10]. Allelopathy is a plant’s effect on another plant [11] manifested as chemical compounds released and diffused in the environment [12,13]. Most allelochemicals are found in plants in an inactive state; they are defene substances against pests [14]. The allelopathic compounds result from hydrolysis processes, from oxidoreduction, methylation, or demethylation. Allelopathic effects occur not only between different plant species, but also between individuals of the same species [15-17]. The main goal of our research experiment was to monitor the behaviour of two plant species with allelopathic properties. *Sorphum halepense* is an herbaceous plant from the gramineae family; it is one of the most damaging crop weeds, particularly in dry areas [18]. It is perennial that produces rhizomes and prefers warm areas. It multiplies through seeds and rhizomes [19]. From a chemical point of view, *S. halepense* plants contain major constituents such as sorgoleone and dihydrosorgoleone. Johnsongrass is one of the plants with significant allelopathic potential [20]. The plant synthesizes sorgoleone benzoquinone which are analogues of these compounds (phytotoxins) [21,22]. This is the reason why Johnsongrass is an inhibitor of other plant’s growth; the effect occurs in the plants’ root hair cells, because they involve the enzyme polyketide synthase that uses fatty acids to form pentadecatrienyl resorcinol intermediaries [23,24]. *D. stramonium* contains a series of alkaloids including atropine (d–1–hiosciamine), hiosciamine, and scopolamine. Biotic processes include both evolutive aspects (adaptation, local extinction) and ecological processes related to the dispersion capacity specific to each plant species, or to the interactions between plant species [25,26]. Allelopathy is a form of plant interaction occurring when a plant intervenes in the growth and development of another plant through chemical inhibitors (toxic substances). Allelopathic substances can be biosynthesised in any plant organ, but they are most frequent in the roots, seeds, and leaves [27,28]. The compounds with allelopathic potential occur virtually in all plant tissues; they either produce their effect through their chemical structure, or are the precursors of other toxic compounds resulted from microbial decomposition, or from physic-chemical alterations [29,30]. This study was conducted to determine allelopathic effects of *D. stramonium.* In this paper, we have analysed the effect of some concentrations of tropane alkaloids extract*,* on Johnsongrass seed germination and seedling growth.

## Results

### Obtaining and identifying alkaloids

*Datura stramonium* has a rich alkaloid spectrum; apart from scopolamine, which is the main alkaloid, it contains hiosciamine, teloidin, etc.

We extracted scopolamine and atropine because, through extraction, hiosciamine (an ester of tropine with (−) tropic acid and with a levogyre character) turns into atropine (a racemic mixture, an ester of tropine with (+) tropic acid). UV/VIS spectrophotometric assay is used for compounds whose structure contains double bonds conjugation.

**Figure 1:**
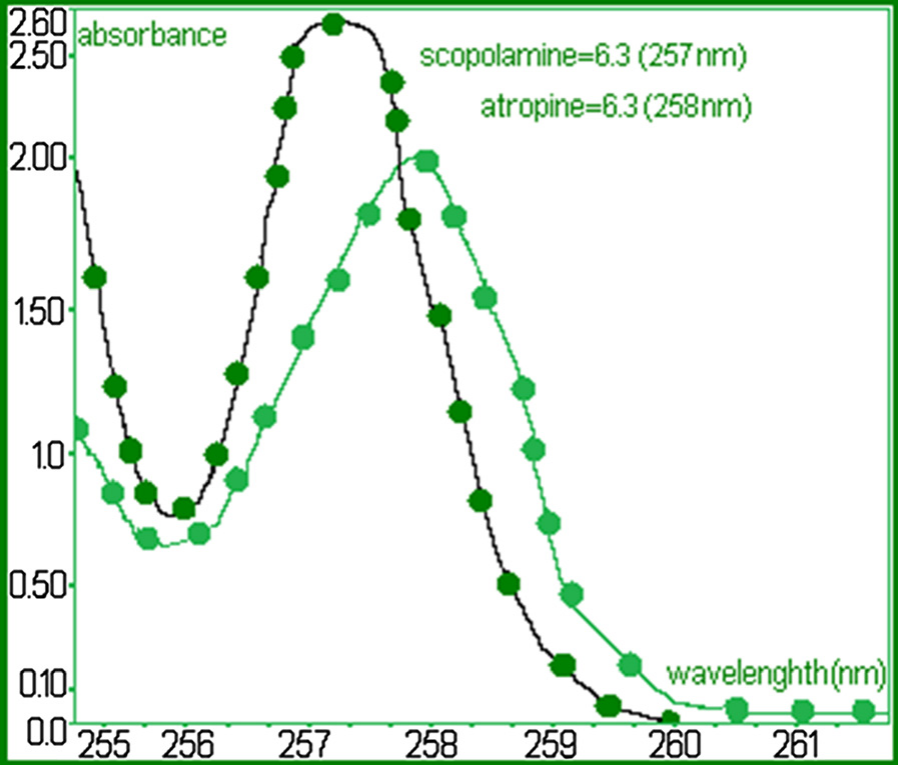
UV–Vis difference spectra of each compound at specific wavelength 6.3 μg atropine (258 nm) and 6.3 μg scopolamine (257 nm).

The concentration values of alkaloids from the *S. halepense* were calculated from the calibration curve obtained using the data in Figure 1.

### Seed germination biotest

The features of the analysed parameters in *S. halepense* seeds treated with different concentrations of scopolamine and atropine extracts from leaves and flowers of *D. stramonium* after 7 days are shown in Table 1.

**Table 1:** **Overall mean values (±S.E.) for various traits of** *** S. halepense *** **seedlings grown under different concentrations**

**Characters**	**Control**	**Concentration of** ***D. stramonium*** **flower extract (μg/g)**			
		**(IV)**	**(III)**	**(II)**	**(I)**
Germination (%)	99.1 ± 0.01	97.3 ± 0.06	98.1 ± 0.02	97.6 ± 0.07	97.4 ± 0.03
Root length (cm)	3.47 ± 0.12	3.38 ± 0.16	3.21 ± 0.18	2.27 ± 0.12	1.96 ± 0.15
Seedling length (cm)	6.29 ± 0.01	6.05 ± 0.01	5.41 ± 0.03	5.26 ± 0.04	4.71 ± 0.02
Seedling/root ratio	1.81 ± 0.08	1.79 ± 0.06	1.68 ± 0.16	2.31 ± 0.33	2.4 ± 0.13
Necrotic area (mm)	0.1 ± 0.001	1.4 ± 0.005	2.3 ± 0.004	6.5 ± 0.02	8.1 ± 0.04
Chlorotic area (mm)	0.1 ± 0.001	1.0 ± 0.017	2.1 ± 0.012	2.3 ±0.015	2.6 ± 0.013
Characters	Control	Concentration of *D. stramonium* leaf extract (μg/g)			
		(IV)	(III)	(II)	(I)
Germination (%)	99.3 ± 0.01	98.2 ± 0.04	98.5 ± 0.08	98.2 ± 0.07	97.8 ± 0.06
Root length (cm)	3.51 ± 0.15	3.49 ± 0.13	3.37 ± 0.12	3.11 ± 0.15	2.71 ± 0.11
Seedling length (cm)	6.24 ± 0.03	4.52 ± 0.02	3.53 ± 0.03	3.12 ± 0.01	2.51 ± 0.05
Seedling/root ratio	1.77 ± 0.2	1.29 ± 0.15	1.04 ± 0.25	1.00 ± 0.06	0.92 ± 0.45
Necrotic area (mm)	0.2 ± 0.002	2.3 ± 0.001	12.5 ± 0.007	14.3 ± 0.005	15.5 ± 0.006
Chlorotic area (mm)	0.1 ± 0.001	6.5 ± 0.02	8.3 ± 0.017	11.7 ± 0.015	12.5 ± 0.013

Mean values (±S.E.).

### Plant growth biotest

The growth degree was significantly higher in the extracts of scopolamine and atropine from leaves than in that from flowers (Table 1). The root system developed by the plants of this species is not that complex: the plants were only able to develop small root systems and most root hairs were absent. The analysis pointed to a significant variation (P < 0.001) in all alkaloid aqueous concentrations: The two alkaloid aqueous extracts (P < 0.05) and the various concentration variants (P < 0.001) had a significant effect on the development of *S. halepense s*eedling development (Table 2). The shortest seedlings length was found in the highest concentration (I) of flower extract from *D. stramonium.* The development of chlorotic areas was visible in the presence of both tropanic alkaloid aqueous extracts; the influence on the growth of *S. halepense* seedlings was significant (P < 0.001). Flower extract concentrations resulted in the formation of considerably larger chlorotic areas compared to the seedlings treated with leaf extract. Increased aqueous extract concentrations resulted in significant effects (P <0.001) on *S. halepense* seedlings (Table 2).

**Table 2:** **Characteristics of** *** S. halepense *** **seedlings grown under different alkaloid concentrations**

**Characters**	**Concentration of** ***D. stramonium*** **extract (μg/g)**							
	**Flower extract**				**Leaf extract**			
Root	(IV)	(III)	(II)	(I)	(IV)	(III)	(II)	(I)
Allelopathic efficiency(AE) (μg/g)	0.42	0.45	0.48	0.61	0.44	0.52	0.68	0.72
Biological absorption coefficient	0.14	0.079	0.049	0.037	0.28	0.171	0.13	0.116
Characters	Concentration of *D. stramonium* extract (μg/g)							
		Flower extract				Leaf extract			
Seedling	(I)	(II)	(III)	(IV)	(I)	(II)	(III)	(IV)	
Allelopathic efficiency(AE) (μg/g)	0.25	0.31	0.37	0.39	0.27	0.35	0.43	0.47	
Biological absorption coefficient	0.46	0.25	0.06	0.053	0.07	0.048	0.039	0.03	

In the presence of flower extract, the allelopathic efficiency of *S. halepense* seedling roots ranged from 0.32, 0.45, 0.48 to 0.61 for the 4 concentrations, while the biological absorption coefficients read 0.14, 0.079, 0.049, and 0.07 for the same concentrations (Table 3). In case of the leaf extract, allelopathic efficiency was higher (0.44, 0.52, 0.68 and 0.72 μg/g, respectively). The same effect was observed in the biological absorption coefficient for which we calculated the following values: 0.28, 0.171, 0.13, and 0.116. For the seedlings of *S. halepense* treated with flower extracts, we measured the following allelopathic efficiency values: 0.25, 0.31, 0.37 and 0.39, which corresponded to the biological absorption coefficients, namely 0.46, 0.25, 0.06 and 0.053. Seedlings treated with leaf extract had the following allelopathic efficiency values: 0.27, 0.35, 0.43 and 0.47, respectively, for which we calculated biological absorption coefficient values of 0.07, 0.048, 0.039, and 0.03.

**Table 3:** **Analysis of variance (mean squares) for various growth features of** *** S. halepense *** **seedlings grown at different alkaloid concentrations**

**Characters**	**MS**_***Datura s***_	**Significance**	**MS**_**Conc**_	**Significance**	**MS**_**Inter**_	**Significance**
Germination (%)	247.80	N.S	38.90	N.S.	141.09	N.S.
Root length (cm)	22.79	***	1.09	**	4.09	***
Seedling length (cm)	4.81	*	9.79	***	1.21	N.S.
Seedling/root ratio	11.98	***	0.82	N.S.	0.67	N.S.
Necrotic area (mm)	0.81	N.S	2.04	***	0.51	N.S.
Chlorotic area (mm)	4.31	***	1.08	***	4.31	***

*,**,***denote significance at P < 0.05, P < 0.01 and P < 0.001 respectively, N.S. = non-significant; MS _*D. stramonium*_ = Mean square _*D. stramonium*_, MS_Conc_ = Mean square alkaloid concentration, MS_Inter_ = Mean square interaction.

### Chlorophyll accumulation decreased in all the variants used in the biotest

**Figure 2:**
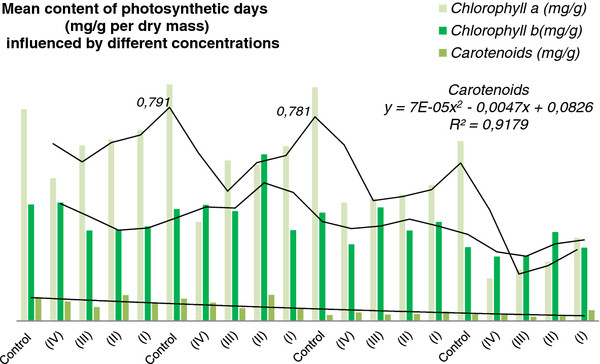
**Mean content of photosynthetic days (mg g**^**–1**^**per dry mass) influenced by different concentrations.**

By monitoring the absorption alterations at 471 nm we assess the activity of chlorophylls, as shown in Table 4. The most used carotenoid and chlorophyll extraction methods use organic solvents. The method allowed the assessment of chlorophyll a (662–664 nm) and chlorophyll b (645 nm), with maximum absorption within the red radiation area; components of the reaction core in photosynthesis systems of maximum absorption pigments in the blue radiation area; wave lengths 471–473 nm, components of the absorption core in photosynthesis systems. The ratio between photosynthesis components showed an increasing trend of chlorophyll b leading to a decrease of the ratio between chlorophylls (Figure 2).

**Table 4:** **Average content of photosynthetic days (mg g**^**–1 **^**per dry mass) in aerial plant parts influenced by different concentrations**

**Days**	**Concentrations**	**Chlorophyll** ***a*** **(mg/g)**	**Chlorophyll** ***b*** **(mg/g)**	**Carotenoids (mg/g)**
1	Control	0.708	0.389	0.075
	(IV)	0.477	0.396	0.065
	(III)	0.587	0.302	0.046
	(II)	0.606	0.305	0.086
	(I)	0.638	0.316	0.061
4	Control	0.791	0.374	0.074
	(IV)	0.331	0.388	0.061
	(III)	0.537	0.367	0.043
	(II)	0.522	0.557	0.087
	(I)	0.584	0.303	0.040
8	Control	0.781	0.362	0.019
	(IV)	0.396	0.256	0.028
	(III)	0.409	0.379	0.021
	(II)	0.423	0.302	0.023
	(I)	0.455	0.331	0.031
12	Control	0.601	0.247	0.023
	(IV)	0.142	0.215	0.022
	(III)	0.172	0.218	0.014
	(II)	0.198	0.297	0.016
	(I)	0.278	0.245	0.036

## Discussion

*S. halepense* seed germination was not affected by the concentration of alkaloids from the tested extracts. Germination started in the 4^th^ day after introducing the seeds in the Petri dishes and ended in the 7^th^ day. By analysing the germination degree per variant we can draw the conclusion that *D. stramonium* extracts added to the medium does not influence germination in either concentration [27]. Germination rate was constant both in the absence and in the presence of these extracts of alkaloids (Table 1).

The highest length values were found in the control samples compared to the samples in which we tested alkaloid aqueous extracts. The lowest value of root length was found in the dilution domain I, with a difference (P < 0.00) of the extract from leaves *D. stramonium*. The values of allelopathic efficiency increased with the decrease of the concentration of extracts used, as well as the values of biological absorption coefficients. The allelopathic efficiency of the *S. halepense* seedlings had the highest value in the concentration domain (I) for both extract types [22,30].

In general, the accumulated amount of carotenes and xanthophylls was low in most variants [24]. The correlation between the low chlorophyll concentrations pointed out the activity of the photosynthesis cycle in which the carotenoids and their monoepoxides act. Pigments can be easily altered or even destroyed. Pigment analysis depending on alkaloid concentration showed that the mean value of chlorophyll evolves following the biapical curve model with the first peak in the 4^th^ day and a second peak in the 8^th^ day [29]. The dependency is expressed as a polynomial model from carotenoids y = 7E-05x^2^ – 0.0047x + 0.0826; and R squared value R² = 0.9179.

*S. Halepense*’s sensitivity was impregnated by allelopathic potential (it contains the cyanogenic glycoside, epimer durine with taxipiline) of the species to accumulate nutrients from the growth medium.

*S. halepense* seeds had a very good germination percentage. Parameter analysis differentiated the two extracts from the point of view of growth and development performances for the concentration variants in the tested culture medium.

Germination biotest results pointed out clearly that the size of extract concentrations had no impact on the germinating capacity of *S. halepense.* The insignificant toxicity effects of tropane alkaloids on the germination of plants seeds suggested that the seeds use their own reserves during the germination process. In exchange, the presence of alkaloids in the culture medium had significant adverse effects on the growth and health of *S. halepense* seedlings. The study pointed out a seedling root length reduction and the development of some chlorotic and necrotic areas.

The presence of the extract of alkaloid from leaves and flowers affected the growth of *S. halepense* seedlings, the reduction of the growth being directly influenced by the increase of the alkaloid concentration (Table 1). The presence of the alkaloids in the culture medium indicates a negative effect on root growth. This suggests that there exists a mechanism preventing the reduction of alkaloid translocation in the root.

The distribution of the two alkaloids extracts was different in cases of the root and the seedling, as shown in Table 2. By analysing biological absorption coefficients we could see that the leaf alkaloid extract concentrates in the roots, while flower extract seemed to diffuse easier in the seedlings. The development of the necrotic areas depended on the concentration. The chlorosis caused by the leaf extract was lower, compared to the chlorosis caused by the use of flower extract. This can be explained by the variable activity of these alkaloid extracts on the chloroplast. Allelopathic efficiency alkaloids extracts on *S. halepense* roots and leaves varied considerably.

## Conclusions

Thus, the goal of this work was to these investigations by germinating, seed and seedling growing, and dosing of photo-synthesis pigments from *S. halepense*, followed by treatment with extract tropane alkaloids. *D. stramonium* plants are fit for the situations in which *S. halepense* becomes a pest, i.e. *S. halepense* should not be occasionally and/or transitorily close to *D. stramonium*. Such researches can stand as a convincing example of feasibility study required by policy-makers in many countries in order to support large-sale studies of this kind and promote and regulate a more nature-inspired approach to agriculture and free larger land surfaces from pesticides. In the study of allelopathy the critical question is whether these compounds are released in the environment in sufficient amounts, and produce, as a consequence, a reaction in other neighbouring bodies.

## Methods

### Plant Extracts

The experiment was carried out in 2010, in the Greenhouse; the biotest aiming to determine seed germination was done in Timisoara Seed Inspectorate’s seed quality control laboratory. Chemical measurements were made in the biochemistry laboratory of the Plant Chemistry and Biochemistry laboratory of the Banat University of Agricultural Science and Veterinary Medicine in Timisoara (Romania).

### Sample Preparation

The vegetal material we used of our biotests consisted in leaves and flowers of *D. stramonium,* from which we prepared aqueous extracts, in the following way: we chopped 250 g of vegetal material and mixed it with 500 mL of water for 30 min; the mixture was then left to macerate for 24 h, and then filtered. The extracts were kept at 4°C. The solutions thus obtained-contained 6.3 μg atropine and 6.3 μg scopolamine-were considered reference solutions.

### Obtaining and identifying alkaloids

Scopolamine and atropine were obtained from an extract from *D. stramonium*.

Alkaloids were marked by characteristic reactions (tested with different reagents). They were identified by the HPLC–UV method of simultaneous determination of scopolamine and atropine in an aqueous extract. We used the pair ion method and diode array detection (DAD) that determines the two substances quantitatively. The HPLC method used a column C_8_ (250 x 4.6 mm) with gradient elution and a column working temperature of 25°C. The mobile phase was constituted of acetonitrile (HPLC grade) 25 % and aqueous solution 75 % (5 mM monohydrate sodium 1–heptansulfonate, pH = 3.5). The compounds detection was performed in UV at λ = 230 nm bandwidth 4 nm/reference λ = 360 nm bandwidth 8 nm/reference. The calibration curve remains linear, within the range 0.13–13.75 μg/mL (scopolamine: r = 0.9951, n = 8) and 0.25–25.5 μg/mL (atropine: r = 0.9999, n = 8) [31].

### Assessment of photosynthesis pigments

In order to determine the absorption spectrum of each pigment, we used a pigment extract in acetone 80 %, from which we separated the pigments. We poured 5 mL of assimilating pigment alcohol extract into a glass Erlenmeyer over which we added 2–3 mL of petroleum ether and a few drops of water, and then we stirred the content. We then separated the phases: the upper part coloured in green, for it contained chlorophyll a and b, whereas the lower part contained yellow carotenod pigments. The photosynthetic pigments (chlorophyll a, chlorophyll b, carotenoids pigments) were assayed using spectrophotometric methods, based on the specific absorption coefficients. Extract extinctions (absorptions) were determined at 440.5, 662 and 646 nm wave lengths with a PG Instruments UV–VIS spectrophotometer using UV WIN 5.05 software. The amount of pigments in the samples was calculated according to the following formulas (1, 2 and 3). Synthesis pigment values were expressed in mg/g dry mass.

(1) $$\text{chlorophyll a }\left(\text{mg}\right)=9.{78\text{ A}}_{662}–0.{99\text{ A}}_{646}$$

(2) $$\text{chlorophyll b }\left(\text{mg}\right)=21.{4\text{ A}}_{646}–4.{65\text{ A}}_{662}$$

(3) $$\text{carotenoid pigments }\left(\text{mg}\right)=4.{69\text{ A}}_{440.5}–0.267\;\left(\text{chlorophyll a}+\text{chlorophyll b}\right)$$

### Bioassays

#### Seed germination biotest

The seeds were checked in order to see whether they comply with all quality standards necessary for germination and growth of seedlings, after which we treated them with a sterilising agent. The seeds of *S. halepense* used in the biotest had a seed germination of 98 %. The seeds were sterilized in sodium hypochlorite 5 % for 10 minutes before use, to avoid fungal contamination. The seeds were well cleaned with ionized water. The extracts from *D. stramonium* were prepared in different concentrations. The concentrations used in these biotests were the following: domain (I)–standard substances (6.3 μg atropine + 6.3 μg scopolamine); domain (II)–obtained through dilution 1:1; domain (III)–obtained through dilution 1:2; domain (IV)–obtained through dilution 1:3. Domain I is similar to the values of alkaloid concentrations from the plant natural extracts tested in the biotest. Thirty Petri plates (8 cm diameter) were washed with deionised water and coated with filter paper for the study of germination. In each Petri plates, we put 20 seeds of *S. halepense* and 20 mL treatment solution and control. The light used to germinate the seeds was cold light emitted by fluorescent tubes with an intensity of 190 μmol/s/m^2^, during a photoperiod of 16 h, for 7 days, until the cotyledons developed completely.

#### Plant growth biotest

The design of the research of the randomised block type contained three separated blocks (replicates). The method starts from a basic plan in which the first replicate is set systematically whereas the next replicates are randomised, for each concentration and type of extract. Each block contains all the variants so that the block corresponds to a replicate, while the number of replicates equals the number of blocks. The variants were set randomly, except for the first replicate (block) in which they were set systematically. Pre-germinated seeds were transferred into laboratory vases. In the vases (30 cm high, 25 cm diameter), we introduced 100 mL extracts of alkaloids, in various concentrations. The vases were filled out with a ½ Murashige-Skoog (MS) medium (simple, with no growth hormones) with sucrose 10 g/L, agar (Sigma) 8 g/L, pH 5.9, 20 mL medium MS/vase, 32 seeds/vase. We kept them in the growth chamber, under stable conditions of temperature and moisture (25 ± 2°C and 45 %). The cultures were grown in vases to ensure the penetration of enough light for optimal physiological processes of assimilation of nutrients from the culture medium, seed germination, photosynthesis, development of organic substances, seedling growth, and further development of the plants. After 12 days, the seedlings were taken out of the solution, washed and measured. Necrotic spots and chlorotic areas were analysed with a Swift M28Z 10x–40x zoom range stereo microscopes. The biological absorption coefficient (BAC) of the fresh state weight was determined with the formula 4:

(4) $$\text{BAC}=\frac{\text{content}\;\text{of}\;\text{the}\;\text{test}\;\text{plants}(\mu g)}{\text{concentrations}\;\text{of}\;\text{aqueous}\;\text{extracts}(\mu g)}$$

Allelopathic efficiency (AE) was calculated according to the equation 5:

(5) $$\text{AE}=\frac{\text{Percentage}\;\text{decrease}\;\text{of}\;\text{dry}\;\text{mass}\;\text{of}\;\text{treated}\;\text{plant}\;\text{as}\;\text{related}\;\text{to}\;\text{control}}{\text{Concentratons}\;\text{of}\;\text{alkaloids}(mL^{-3})}$$

#### Statistical analysis

All values were expressed as mean ± S.D. Statistical significance was evaluated by Students–"t" test (the conditional significance level of Student's two-sided *t*-test and the coverage of the related confidence intervals) at 5 % level of significance (p < 0.05) and analysis of variance (ANOVA) is a collection of statistical models, and their associated procedures, in which the observed mean squares.

## Competing interests

The author declares that they have no competing interests.
